# Fractalkine Signaling Regulates the Inflammatory Response in an α-Synuclein Model of Parkinson Disease

**DOI:** 10.1371/journal.pone.0140566

**Published:** 2015-10-15

**Authors:** Aaron D. Thome, David G. Standaert, Ashley S. Harms

**Affiliations:** Center for Neurodegeneration and Experimental Therapeutics, Department of Neurology, The University of Alabama at Birmingham, Birmingham, Alabama, United States of America; Emory University, UNITED STATES

## Abstract

**Background:**

Parkinson disease (PD) is a progressive neurodegenerative disorder characterized by loss of dopamine neurons in the substantia nigra pars compacta (SNpc) and widespread aggregates of the protein alpha-synuclein (α-syn). Increasing evidence points to inflammation as a chief mediator; however, the role of α-syn in triggering and sustaining inflammation remains unclear. In models of Alzheimer’s disease (AD), multiple sclerosis (MS) and neurotoxin models of PD, the chemokine CX3CL1 (fractalkine) and its receptor (CX3CR1) have important roles in modulating neuroinflammation.

**Methods:**

To examine the role of fractalkine signaling in α-syn-induced-neuroinflammation and neurodegeneration, we used an *in vivo* mouse model in which human α-syn is overexpressed by an adeno associated viral vector serotype 2 (AAV2) and *in vitro* phagocytosis and protein internalization assays with primary microglia treated with aggregated α-syn.

**Results:**

We observed that loss of CX3CR1 expression led to a reduced inflammatory response, with reduced IgG deposition and expression of MHCII 4 weeks post-transduction. Six months post transduction, AAV2 mediated overexpression of α-syn leads to loss of dopaminergic neurons, and this loss was not exacerbated in animals with deletion of CX3CR1. To determine the mechanism by which CX3CR1affects inflammatory responses in α-syn-induced inflammation, phagocytosis was assessed using a fluorescent microsphere assay as well as by microglial uptake of aggregated α-syn. CX3CR1-/- microglia showed reduced uptake of fluorescent beads and aggregated α-syn.

**Conclusion:**

Our results suggest that one mechanism by which CX3CR1-/- attenuates inflammation is at the level of phagocytosis of aggregated α-syn by microglia. These data implicate fractalkine signaling as a potential therapeutic target for regulating inflammatory response in α-syn models PD.

## Introduction

Parkinson disease (PD) is a common neurodegenerative movement disorder characterized by a progressive loss of dopamine producing neurons in the substantia nigra pars compacta (SNpc) and widespread intracellular aggregates of the protein alpha-synuclein (α-syn). This protein is the principal component of Lewy bodies and Lewy neurites, the pathological hallmark of PD. α-syn has been genetically implicated through studies of familial PD. Mutations and multiplications in the SNCA locus convey risk for sporadic PD. [1,2]. Genome-wide association studies (GWAS) have also linked the SNCA locus to PD susceptibility in sporadic disease [3] Together, these observations point to a central role for α-syn in the etiology of PD, although the mechanisms by which α-syn initiates the disease and subsequent neurodegeneration remain uncertain.

Increasing evidence points to a key role of neuroinflammation in the pathogenesis of PD[4–6]. Analysis of post mortem brain and cerebral spinal fluid from PD patients show increased pro-inflammatory cytokines such as TNF, IL-1β, IL-6, and IFN-γ [7,8]. Extensive reactive microgliosis [6,9] and T cell infiltration [10] are present indicating a strong pro-inflammatory immune response. In addition, GWAS have implicated polymorphisms in the HLA-DR locus, also known as MHC class II, in late-onset PD [11]. MHC class II is expressed on antigen presenting cells including microglia, and is critical for mounting an adaptive immune response by presenting antigen to CD4+ T cells.

All of the inflammatory features found in human PD are also observed in animal models. Viral overexpression, transgenic, and neurotoxin models of PD in rodents and non-human primates recapitulate the reactive microgliosis, elevated pro-inflammatory cytokine expression, lymphocyte infiltration, and loss of TH+ cells in the SNpc [12,13]. Our lab has previously shown that modulation of inflammation-related components can attenuate or block the inflammatory response to α-syn. Specifically, Cao et al. demonstrated the importance of Fc receptors in the phagocytic pathway in microglia [14] while Harms et al. showed that subsequent processing and presentation of antigen by MHCII is important for α-syn induced inflammation and neurodegeneration [15].

CX3CL1 (fractalkine) and its specific receptor, CX3CR1 have important roles in modulating inflammation in the CNS [16]. Found abundantly on membranes of neurons and endothelial cells, fractalkine functions as a chemokine by signaling to its receptors found on microglia in the brain and monocytes, dendritic cells, and natural killer cells in the periphery to initiate chemotaxis and activation of these cells [17,18]. It has been shown in the CNS that fractalkine signaling guides microglial migration during development and dictates microglial effector functions [19,20]. Fractalkine signaling through CX3CR1, in turn, regulates the neurotoxic properties of microglia under inflammatory conditions by helping the cells maintain a quiescent state [21]. Knockout of CX3CR1 in neurotoxin models of PD leads to enhanced neurotoxic effects. In a LPS mouse model, CX3CR1 knockout led to increased nigral neuron degeneration associated with an enhanced IL-1β cytokine response. In an 1-methyl-4-phenyl-1,2,3,6-tetrahydropyridine (MPTP) neurotoxin model of PD, CX3CR1 knockout exacerbated inflammation and neurodegeneration [22]. In α-syn models, over-expression of fractalkine ligand has been shown to protective[23], but the effects of CX3CR1 knockout have not been examined.

In this study, we used adeno-associated virus (AAV) overexpression of α-syn *in vivo* and primary mouse microglia *in vitro* to study the role of fractalkine receptor in modulating α-syn-induced inflammation and neurodegeneration. In contrast to the effects observed in neurotoxin models, we find that knockout of CX3CR1 attenuates the inflammation and fails to exacerbate neurodegeneration observed with AAV2-SYN-mediated overexpression. In examining the microglia *in vitro*, we find that CX3CR1-/- impaired or reduced uptake of either synthetic fluorescent microspheres or aggregated α-syn. These data suggest that CX3CR1 is important in disease progression of synucleinopathies, and could potentially be a target for neuroprotective therapies for PD.

## Methods and Materials

### Animals

C57BL/6 (catalog # 000664) and CX3CR1-/- mice (B6.129P-*Cx3cr1* ^*tm1Litt*^ */J* (catalog # 005582) Cardona AE et al., 2006) maintained on a congenic background were used for these studies and were obtained from Jackson Laboratories (Bar Harbor, Maine). The locus for CX3CR1 was disrupted via insertion of the sequence encoding green fluorescent protein (GFP), replacing the first 390 bp of the coding exon (exon 2). The deleted region includes an amino-terminal portion of the protein that is critical for interaction with endogenous fractalkine ligand, CX3CL1. RIKO mice show abnormal microglial cell physiology including microglial migration during development [22]. These mice show increased susceptibility to experimental autoimmune encephalomyelitis and increased neuronal apoptosis following LPS exposure [22].

### AAV2 Virus construction and purification

Construction and purification of the rAAV vectors rAAV-CBA-IRES-EGFP-WPRE (CIGW) and rAAV-CBΑ-SYN UCLEIN- IRES-EGFP-WPRE (CSIGW) are described in previous publications [24–26].

### Stereotaxic Surgery

Male C57BL/6 (WT) and CX3CR1 knockout mice (8–12 weeks of age) were anesthetized with isoflurane and unilaterally injected with 2uL of AAV2-SYN of AAV2-GFP (4.0x10^12^ viral genome/mL diluted in sterile PBS) into the right SNpc. Co-ordinates were anterior- posterior -3.2 mm from bregma, medio-lateral -1.2 mm from midline, and dorso-ventral -4.6 mm from dura. All research conducted on animals was approved by the Institutional Animal Care and Use Committee (IACUC) at the University of Alabama at Birmingham.

### Immunohistochemistry

At 4 weeks and 6 months post-transduction, animals were deeply anesthetized, sacrificed, and brains were removed and processed. Briefly, animals were transcardially perfused with heparinized 0.01M phosphate-buffered saline (PBS) pH 7.4 followed by 4% paraformaldehyde and then cryoprotected in 30% sucrose solution in PBS. Brains were frozen on dry ice and cryosectioned coronally on a sliding microtome (cut thickness: 40um); sections were collected serially throughout the striatum and SNpc, placed into tissue collection solution (50% glycerol in 0.01M PBS), and stored at -20C for immunohistochemical analysis.

Fluorescent analyses of free-floating sections were labeled as previously described. [15] Sections were labeled with anti-MHCII (M5/114.15.2, eBiosciences, 1:100), anti-GFP (Rockland, Gilbertsville, PA, 1:1000), or anti-TH (Millipore, 1:2000) overnight at 4°C. Respective Alexa-conjugated secondary antibodies diluted at 1:1000 (Invitrogen) were used at room temperature for 2.5 hours. IgG staining utilized a Cy3 conjugated goat anti-mouse IgG secondary (JacksonImmunoResearch, 1:500). Sections were mounted onto plus coated glass slides and cover slips were added using Vectashield Hard Set mounting medium.

For diaminobenzadine (DAB) staining of free-floating sections, sections were washed and stained with Tris-buffered saline (TBS) at pH 7.4 and labeled as previously described [15]. Briefly, anti-MHCII (M5/114.15.2, eBiosciences, 1:100) or anti-TH (Millipore, 1:2000) was diluted in TBS-triton (TBST) + 1% normal serum and incubated overnight at 4C. Appropriate biotinylated secondary (Vector Laboraties, Burlington, CA, 1:500) was added in TBST plus 1% serum and incubated for 2 hours at room temperature. R.T.U. Vectastain ABC reagent (Vector Laboratories, Burlington, CA,) was added according to manufacturer’s instructions and incubated for 1 hour at room temperature. A DAB kit from Vector laboratories was used to develop staining according to manufacturers instructions with or without the addition of nickel. Sections were mounted onto plus coated glass slides and cover slips were added using Vectashield Hard Set mounting medium.

For TH neuron quantification using unbiased stereology, free floating sections were stained with anti-Tyrosine Hydroxylase (TH) (Millipore, 1:2000), coded, and analyzed with an Olympus BX51 with MicroBrightfield software (MicroBrightfield Inc., Williston, VT). A total of 5 sections covering the rostro-caudal extent of the SNpc of ipsilateral and contralateral to the injection site were quantified using the optical fractionator method and the StereoInvestigator software. Neurons that stained positive for TH were counted and weighted section thickness was used to correct for variations in tissue thickness.

### Imaging and Quantification

Images were captured using a Leica TCS-SP5 laser scanning confocal microscope. Fluorescent images for MHCII and IgG were exported and processed using Adobe Photoshop. For quantification of MHCII Ni-DAB staining, slides were observed using a Nikon Eclipse E800M microscope. Slides were coded and scored using a numerical scale of 0 (no staining) to 4 (most intense staining) by a single observer blind to the treatment paradigm. Only MHCII staining surrounding the SNpc via TH positive neurons (DAB-brown) were scored while non-specific staining surrounding the needle tract was ignored. Scores were obtained from 5–6 mice per group. The groups were statistically analyzed using Kruskal-Wallis and Dunn’s multiple comparisons test (p = 0.0146).

### Primary Microglia Cultures

Primary murine microglia were isolated from postnatal day 0–2 pups according to previously published protocols [27] with a few modifications. Briefly, brains were isolated, meninges removed and dissociated for 10 minutes at 37C with frequent agitation. Mixed glial populations were filtered though 0.2 micron filter and plated in T75 flasks in DMEM/F12 supplemented with 20% heat inactivated fetal bovine serum (Sigma-Aldrich, St. Louis, MO), 1% penicillin/streptomycin, 1% L-Glutamine (Sigma-Aldrich, St. Louis, MO) and 10ng/mL granulocyte monocyte colony stimulating factor (GM-CSF, PeproTech, Rocky Hill, NJ) for 10–14 days. Microglia were isolated from the astrocyte bed by mechanical shaking at 195 rpm for 1 hour at 37C.

### α-syn Treatment and Fluorescent Microsphere assay

Microglia were plated in chamber slides (Lab-Tek II Chamber Slides, Rochester, NY) at 100,000 cells per well with serum free media. Purified recombinant human α-syn (r-peptide, Athens, GA) was resuspended and incubated at 37C with constant agitation (500rpm) for 7 days as previously described [14]. Prior to assays, microglia are allowed to settle onto chamber slide for 2 hours. After incubation for 24 hours, Nile Red fluorescent microspheres (Invitrogen) washed in PBS containing 1mg/mL BSA were added to chambers for 30 minutes. Cells were then washed with PBS 3 times and fixed with 4% paraformaldehyde in 0.01 PBS. Cells were cover slipped with Vectashield Mounting Medium (Vector Laboratories, Burlingame, CA). Quantification of microspheres was performed using differential interference contrast (DIC) microscopy on the Leica TCS-SP5 laser scanning confocal microscope to count the number of fluorescent spheres within microglia. Approximately 10 random fields containing 5–8 microglia per field were counted and tabulated. Genotypic differences in phagocytosis of the spheres were also analyzed between WT and CX3CR1-/- mice.

### Antigen processing and presentation

Microglia were cultured as previously described in the methods from both the WT and CX3CR1-/- mice. Microglia were plated in chamber slides (Lab-Tek II Chamber Slides, Rochester, NY) at 100,000 cells per well with serum free media. Following 24 hours, cells were treated with 500nM aggregated α-syn for 4 hours followed by 5μL DQ Ovalbumin Red BSA (Molecular Probes, Eugene, OR) was added for 90 minutes. Cells were then washed with PBS and fixed using 1% PFA. Imaging was performed using the Leica TCS-SP5 laser scanning confocal microscope and quantified using ImageJ software as previously described[15].

## Results

### CX3CR1 -/- attenuates α-syn-induced MHCII activation

**Fig 1:**
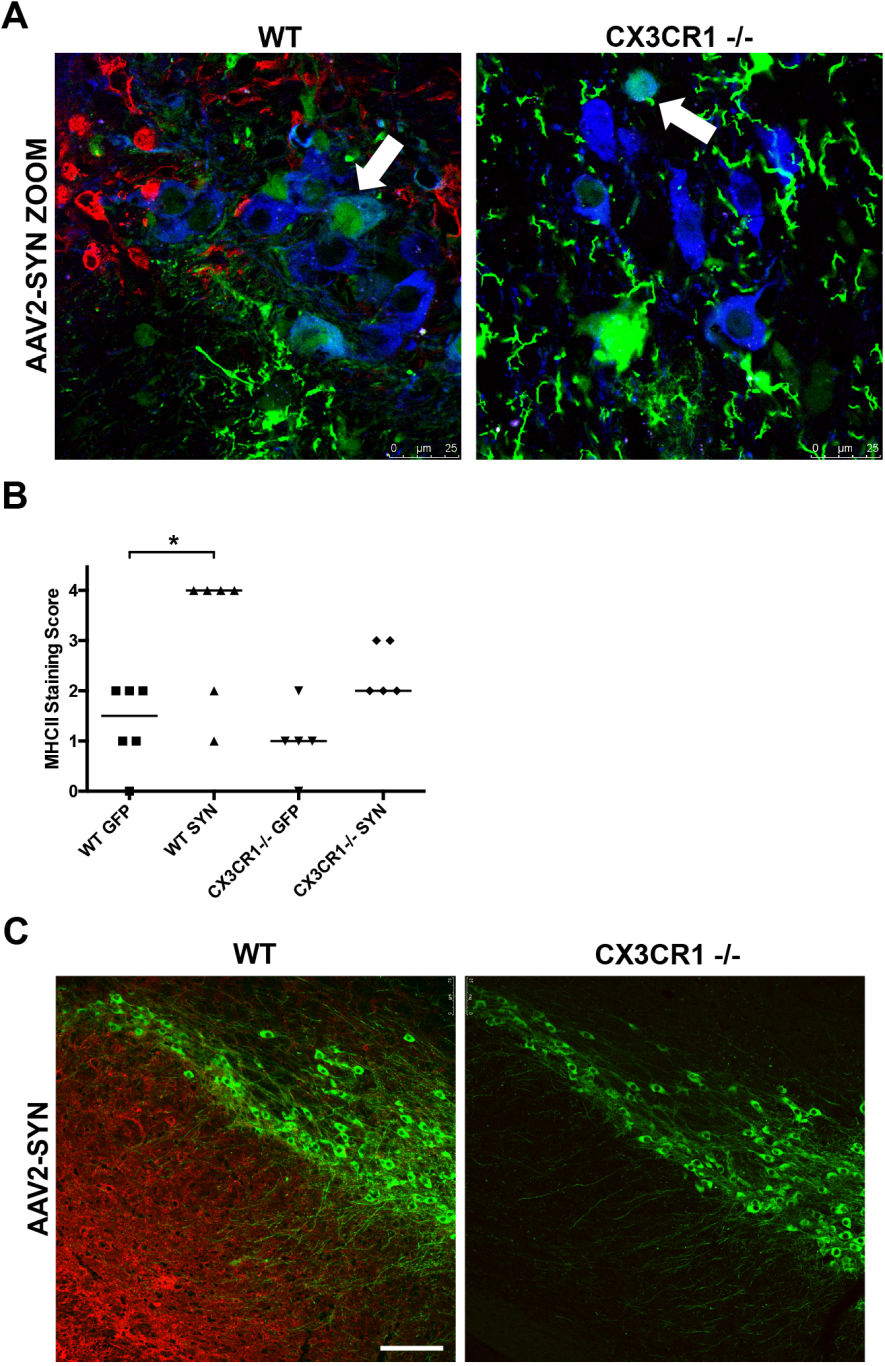
MHCII expression and IgG deposition is attenuated in CX3CR1-/- mice following α-SYN overexpression. (A) MHCII expression (red) in the ipsilateral SNpc (TH blue) following 4 week AAV2-SYN transduction (green). Scale bar = 25um. (B) Quantification of MHCII expression at 4 weeks post transduction of AAV2-GFP or AAV2-SYN in WT and CX3CR1-/- mice. Ipsilateral SNpc sections were rated by a reader blinded to the treatment group. Blinded ratings displayed as medians with ranges with a rating of 4 being highest MHCII expression. n = 5-6/group, p = 0.0146 using Kruskal-Wallis test with Dunn’s multiple comparison test. (C) IgG deposition (red) in ipsilateral SNpc (TH green) following 4 weeks AAV2-SYN transduction in WT and CX3CR1-/- mice. Scale bar = 125um. Images taken using Leica Microsystems TCS SP5 Visible-Upright Confocal microscope.

We have previously shown that overexpression of full-length, human α-syn via AAV2 in the mouse SNpc elicits a neuroinflammatory response which includes an increase in MHCII expression as well as a robust deposition of IgG in the ipsilateral SNpc as early as 4 weeks post transduction [15,24,26]. To determine the role of fractalkine signaling in α-syn-induced neuroinflammation, we administered a single, unilateral stereotaxic injection of recombinant AAV encoding human, full-length α-syn (AAV2-SYN) or AAV2-GFP into the right SNpc of 8–12 week old male C57BL/6 WT and CX3CR1-/- mice (n = 6 animals per group). At four weeks post-transduction, we observed strong expression of both AAV2-SYN and AAV2-GFP in TH+ nigral neurons (Fig 1A, arrow inset) accompanied by increased expression of MHCII in AAV2-SYN-transduced animals but not in the AAV2-GFP-transduced controls. In contrast, the CX3CR1-/- animals show no increase in MHCII expression after AAV2-SYN transduction. (Fig 1A). This was confirmed using DAB staining and rating by an investigator blinded to the treatments (rated 0–4 in staining intensity) (Fig 1B). The blinded rating confirmed reduced expression of MHCII in the CX3CR1-/- animal (p = 0.0146, Kruskal-Wallis test (n = 6/group) with a Dunn’s multiple comparison).

### CX3CR1 -/- blocks IgG deposition in response to α-syn

Deposition of IgG is a component of the inflammatory response to AAV2-SYN, and also observed in human PD [28]. Four weeks post-transduction with AAV-SYN, we examined the amount of IgG deposition in and around the ipsilateral SNpc. In CX3CR1-/- animals, we found a marked decrease in the amount of IgG deposited in the ipsilateral hemisphere at four weeks post transduction when compared to WT animals (Fig 1C).

### CX3CR1 -/- does not exacerbate α-syn-induced neurodegeneration

**Fig 2:**
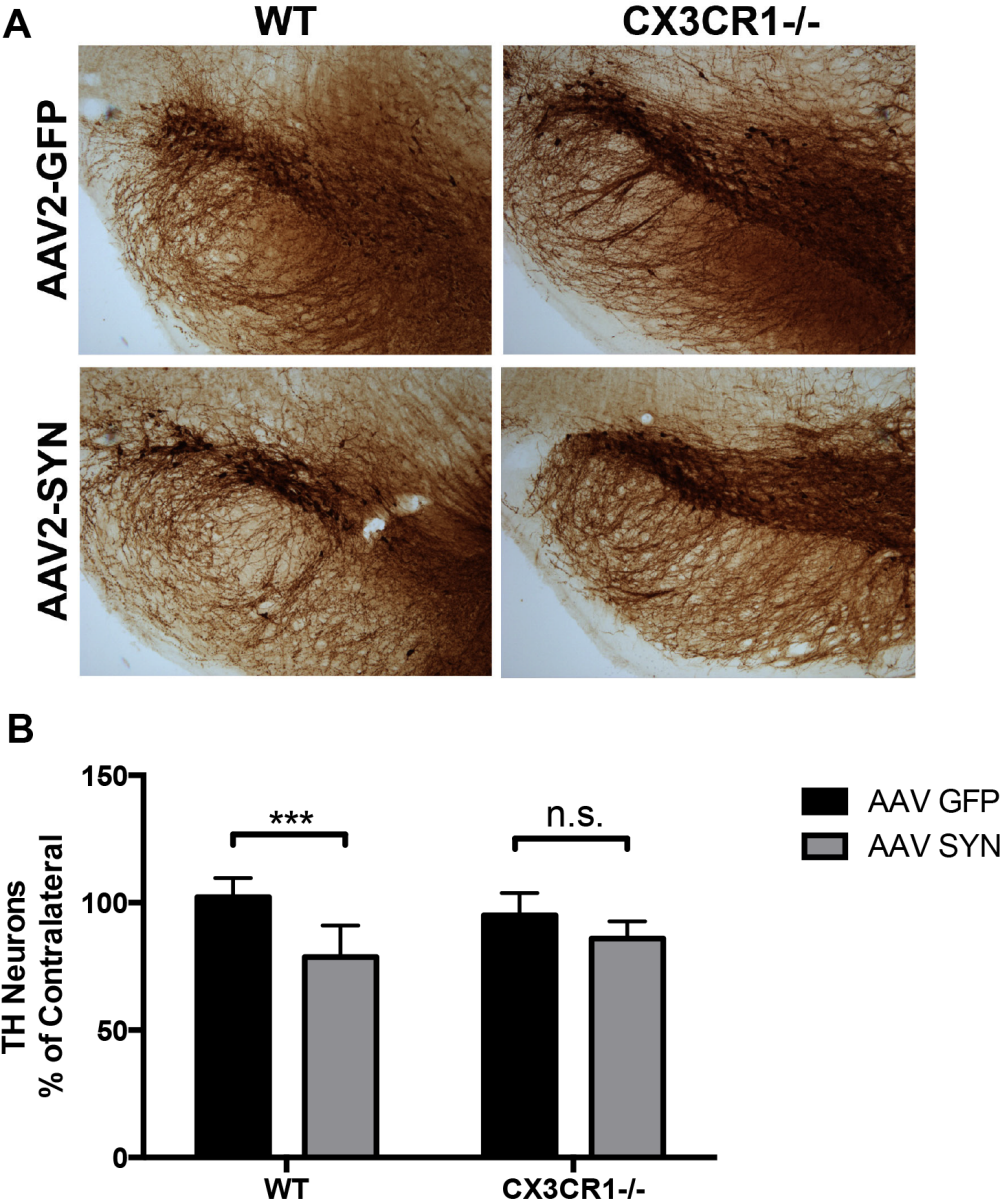
The effect of CX3CR1-/- on α-syn-induced TH+ neuron loss at 6 months post transduction of AAV2-SYN. (A) TH+ neuron chromogen staining (Ni-DAB) of the ipsilateral SNpc 6 months post transduction in AAV2-GFP or AAV2-SYN injected WT and CX3CR1-/- mice. (B) Unbiased stereological cell counts of TH+ neurons in the ipsilateral SNpc from WT and CX3CR1-/- mice at 6 months post transduction of AAV2-GFP or AAV2-SYN. Unbiased cell counts are reported as a percent of the contralateral side. Two-way ANOVA with Sidak’s multiple comparisons test (p<0.001) (n = 7–9 mice per group). Images captured using Nikon Eclipse E800 microscope.

Using the AAV-SYN model, we as well as others have found that viral-mediated overexpression of α-syn in mice results in a loss of approximately 30% of TH-immunopositive cells in the ipsilateral SNpc at six months post transduction. In order to determine whether or not CX3CR1 was involved in the neurodegenerative process in our model, we injected 8–12 week-old male C57BL/6 mice, including both WT and CX3CR1-/-, with a single, unilateral, stereotactic injection of AAV2-SYN and AAV2-GFP control virus into the right SNpc (n = 7–9 animals per group). At 6 months post transduction, TH+ nigral cell number was assessed by unbiased stereology. Our results indicate that CX3CR1-/- fails to exacerbate α-syn-induced dopaminergic cell loss in our model where it has previously exacerbated TH+ cell loss in neurotoxin models of PD. A 23.5±4.6% decrease (ipsilateral counts as a percentage of contralateral) in the number of TH+ neurons in the SNpc was found when WT animals were injected with AAV2-SYN (Fig 2A and 2B, p<0.05 vs. AAV-GFP control). In the CX3CR1-/- animals we saw a trend for neuroprotection (9.12%±2.3) which was not statistically significant when compared to the AAV2-GFP control vector. (S1 Fig). Statistics were described using Two-way ANOVA with Sidak’s multiple comparisons tests. A significant difference was seen between WT AAV-GFP and AAV-SYN treatments while no significant difference was seen between CX3CR1-/- AAV-GFP and AAV-SYN treatments.

### CX3CR1 is important for normal microglial phagocytic responses *in vitro*

**Fig 3:**
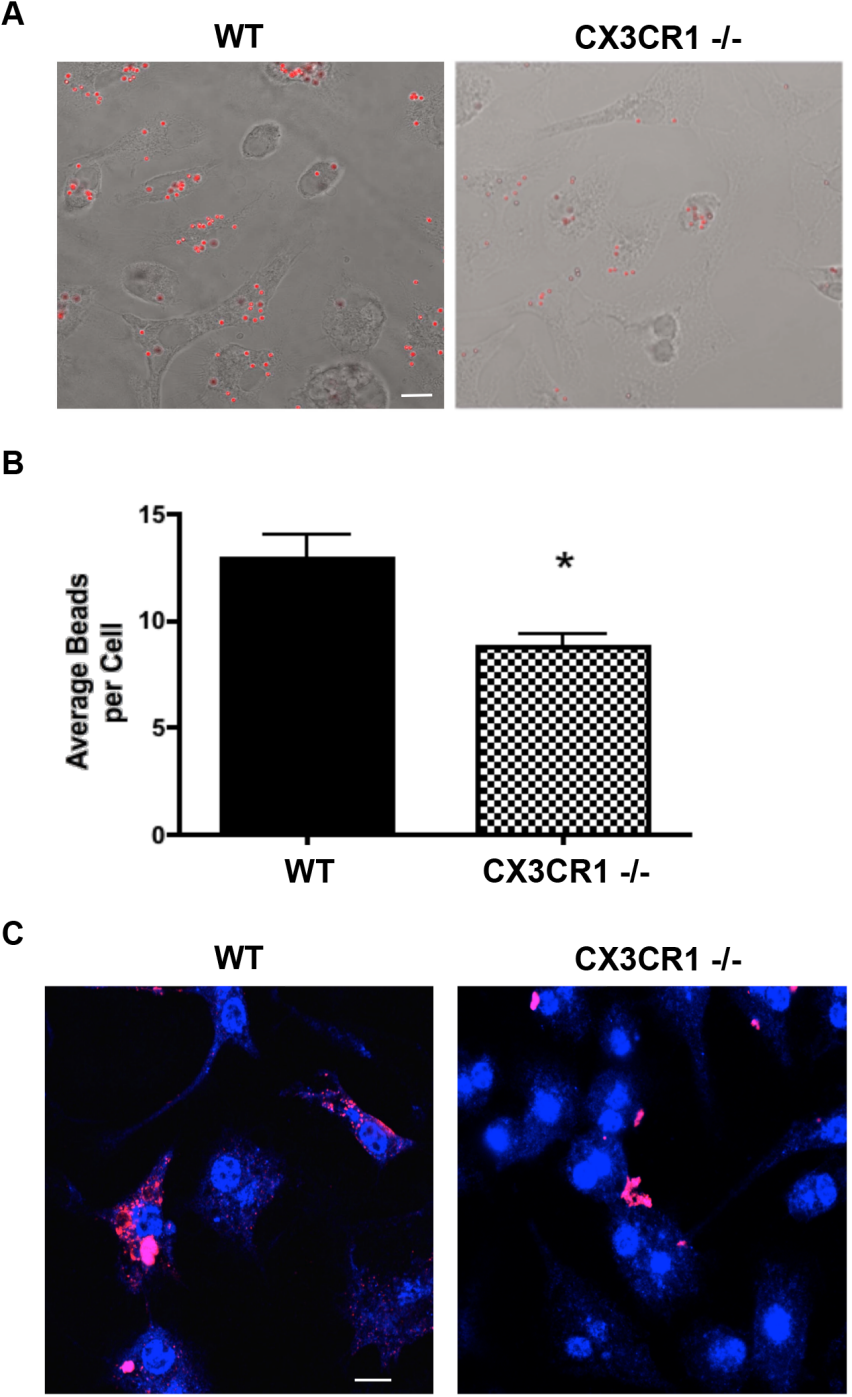
CX3CR1-/- primary microglia show decreased internalization of α-syn and phagocytosis *in vitro*. (A) DIC and fluorescent images from primary microglia isolated from WT and CX3CR1-/- following exposure to Nile Red fluorescent microspheres for 30 minutes. Scale = 10um. (B) Quantification of the average number of beads internalized per microglia and statistically quantified using Student’s t-test (p = 0.0019). (C) Internalization of aggregated α-SYN (anti-Syn204, red) in primary microglia (IBA1 blue) from WT and CX3CR1-/- mice. Scale = 10um. Images taken using Leica Microsystems TCS SP5 Visible-Upright Confocal microscope.

The microglial phagocytic response in the CNS is crucial for proper uptake and disposal of foreign antigens and peptides. To determine the role of fractalkine signaling on microglial phagocytosis *in vitro*, we isolated primary microglia from both WT and CX3CR1-/- mice and plated them at 100,000 cells per well in chamber slides. Using uptake of fluorescent beads, we observed a reduced phagocytic capacity in the CX3CR1-/- microglia compared to the WT microglia (Fig 3A and 3B), in concurrence with previous publications [29]. To study the effect of CX3CR1-/- on phagocytosis of α-syn, aggregated α-syn was added to cultures at 500nM and allowed to incubate for 4 hours. The cells were then fixed with 1% PFA and internalization was analyzed by immunocytochemistry. Fluorescence analysis of microglial cells showed internalization of aggregated α-syn by WT cells, which resulted in smaller, punctate inclusions within the microglia. In contrast to these findings, the CX3CR1-/- microglia displayed reduced capacity to internalize the protein leaving aggregated α-syn at the cell surface (Fig 3C). Larger aggregates were found outside the cells with few to no smaller, punctate inclusions within the cells themselves.

**Fig 4:**
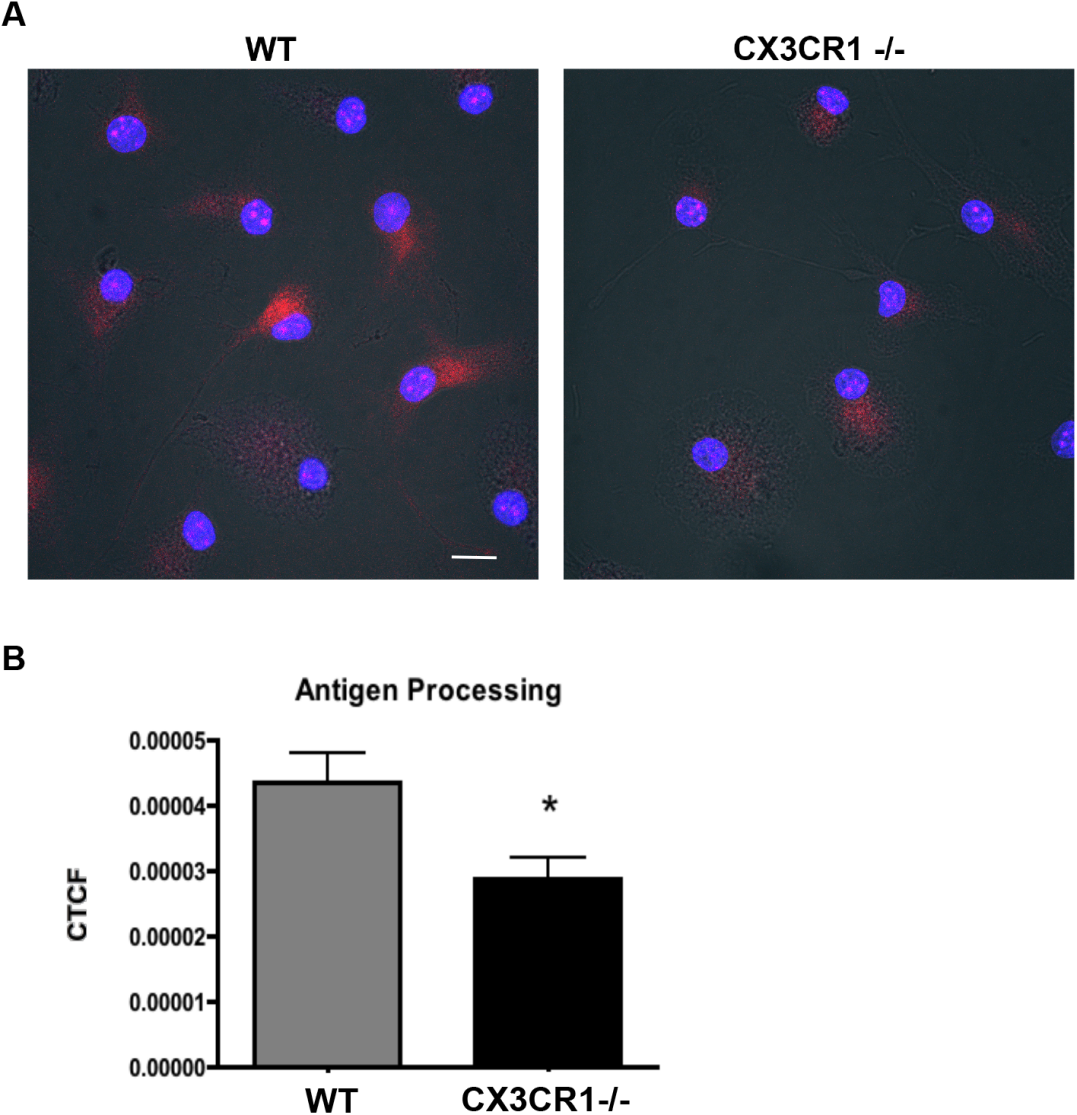
CX3CR1-/- primary microglia show decreased antigen processing in *in vitro*. (A) Antigen processing of DQ Ovalbumin (red) is decreased in CX3CR1-/- primary microglia compared to WT primary microglia following a 30 minute treatment. Scale = 10um. (B) ImageJ quantification of corrected total cell fluorescence (CTCF) of processed DQ ovalbumin (red). Using chamber slides for the experiment, each chamber is used as an independent variable with 4–5 pictures per chamber including 5–8 microglia per picture. 4 chambers (1 slide) used per treatment group and experiment replicated 3 times. Students t-test used for statistical analysis (p = 0.0358). Images taken using Leica Microsystems TCS SP5 Visible-Upright Confocal microscope.

To determine whether direct microglial uptake of α-syn effects the pro-inflammatory response, we used a similar approach to examine the effect of CX3CR1-/- on antigen processing and presentation. We isolated primary microglia from both WT and CX3CR1-/- mice and pretreated with 500nM aggregated α-syn for 4 hours prior to addition of DQ-Ovalbumin Red. DQ-Ovalbumin Red consists of a self-quenching conjugate of ovalbumin that fluoresces upon proteolytic degradation in the lysosome. In WT microglia, the addition of aggregated α-syn led to an increase in fluorescence, indicating cleavage of the DQ-ovalbumin substrate. This signal was reduced by 34% decrease in the CX3CR1-/- microglia reflecting a reduction in the processing activity in response to aggregated α-syn. (Fig 4A and 4B)

## Discussion

In this study we found that deletion of the fractalkine receptor in mice attenuated the α-syn-induced pro-inflammatory response. *In vivo*, this decrease in inflammation is associated with a marked decrease in microglial MHCII expression and a reduction of IgG deposition in the SNpc four weeks post-transduction of AAV2-SYN. We also found that deletion of the fractalkine receptor failed to exacerbate α-syn-induced TH+ neuron loss at 6 months as observed in neurotoxin models of PD. This marked reduction of inflammation may be related to decreases in microglial effector function: using both fluorescent microspheres and aggregated α-syn *in vitro*, we observed a decreased capacity of CX3CR1-/- microglia to internalize these substrates, and reduced α-syn-stimulated antigen processing and presentation indicating a role for CX3CR1 in α-syn internalization and initiation of the pro-inflammatory response. Similar results of CX3CR1 deletion on phagocytosis of protein aggregates have been reported by others [29].

The role of fractalkine signaling within different neurodegenerative rodent models seems to depend greatly on the nature of the model and the events which trigger the degenerative process. In 1-methyl-4-phenyl-1,2,3,6-tetrahydropyridine (MPTP) and 6-hydroxydopamine neurotoxin models of PD, a robust neuroinflammatory response along with extensive neurodegeneration is observed[13]. Deletion of CX3CR1 exacerbates MPTP-induced neuroinflammation and dopamine cell loss in the SNpc in these models [22]. Consistent with this idea, administration of soluble fractalkine ligand reduces neurodegeneration in the 6-hydroxydopamine (6-OHDA) neurotoxin model of PD [30]. Administration of soluble fractalkine ligand also attenuates dopaminergic cell loss and reactive microgliosis resulting from MPTP exposure [31]. It has been suggested that these effects result from the ability of fractalkine ligand to alter the phenotype of macrophages from an M1 or pro-inflammatory phenotype, to an M2 or anti-inflammatory phenotype [32].

Studies involving fractalkine signaling in proteinopathy models, where aggregated or misfolded proteins are the triggering factor, have yielded different results. In models of Alzheimer disease produced by overexpression of APP/PS1 or human APP, CX3CR1 knockout results in decreased levels of pro-inflammatory cytokines Tumor Necrosis Factor (TNF) and monocyte chemotactic protein 1 (CCL2) but increased Interleukin 1-beta (IL1β) [29,33,34]. Our results are very much comparable to these: we find that CX3CR1 knockout in an α-syn model of degeneration attenuates inflammation. Recently, a study described the effects of administration of fractalkine ligand in the AAV2-SYN α-syn rat model of PD[35]. They reported that addition of fractalkine ligand in this model reduces α -syn-mediated neurodegeneration in the SNpc [35]. Interestingly, this protection was observed even though the microglia still showed robust MHCII activation.

Putting these various observations in diverse systems together begins to provide insight in to the complexity of fractalkine signaling and its role in neurodegenerative disease. In models mediated by cellular toxins such as MPTP and 6-OHDA, enhancing the expression of fractalkine ligand provides protection, while knockout of the CX3CR1 receptor worsens pathology[21,22]. It seems likely that this may reflect a direct effect of fractalkine on the inflammatory and cytotoxic properties of activated microglia. On the other hand, in models triggered by protein aggregation, the effects of altered fractalkine signaling are different. In both the AD models described above and the data we have presented on the AAV-SYN mouse model of PD, knockout of CX3CR1 reduces inflammation. We suggest that this may arise from an upstream effect, the inhibition of phagocytosis produced by knockout of CX3CR1. Since this is a necessary initiating step for proteinopathy models, the impairment of phagocytic activity may be the dominant factor in these models, leading to a reduction in inflammation.

The recent study of Nash et al. using fractalkine ligand in an AAV-SYN model may provide some additional insight, but there are issues which require these comparisons to be made cautiously. Most importantly, the Nash et al. study was conducted in rats, while all the other studies described were conducted in mice, and it is possible that species difference is a contributor. A second issue relates to the spatial distribution of the effects; while the knockout is global, affecting all monocytes both within and outside of the brain, the overexpression of fractalkine in the rat was induced locally. Lastly, and we believe most likely, fractalkine may have multiple effects on the pathway between protein overexpression and neurodegeneration. In animals with deletion of CX3CR1, the dominant effect may be impairment of phagocytosis, an upstream event which prevents inflammation. Overexpression of the ligand appears to act far downstream, as demonstrated by the persistence of enhanced MHCII expression despite reduced neurodegeneration when AAV-SYN and fractalkine ligand are co-expressed.

In the field of PD research there is a need for the development of neuroprotective strategies to delay the onset or progression of the disease. Fractalkine signaling appears to have potent effects on PD inflammatory models, but these effects are complex and depend on the nature of the triggering event. Indeed, depending on model is possible to demonstrate both pro- and anti-inflammatory effects of fractalkine signaling. In the context of human disease, the most important issue may prove to be the timing of fractalkine signaling with respect to the degenerative process. Early in PD, blockade of fractalkine may slow disease initiation by interfering with phagocytosis. In established PD, the ability of fractalkine ligand to modulate the inflammatory state of monocytes may be the more important. It will be important to understand more about the state of fractalkine signaling in human disease in order to use this information effectively in the search for a neuroprotective treatment.

## Conclusion

We conclude that CX3CR1 knockout decreases phagocytosis in microglia resulting in decreased inflammation associated with α-syn internalization. The decreased internalization of aggregated α-syn results in attenuated antigen processing and presentation in microglia. In vivo, these deficits result in a decrease in inflammation marked by a decrease in MHCII expression and IgG deposition. As a result of the attenuated immune response, we also found that deletion of the fractalkine receptor, CX3CR1, did not exacerbate α-syn-induced TH+ neuron loss in the ipsilateral SNpc at 6 months post transduction as reported in neurotoxin models of PD. These data implicate fractalkine signaling as a potential therapeutic target for regulating the inflammatory response in α-syn models PD.

## Supporting Information

S1 Fig Contralateral TH Neuron Cell Counts in WT and CX3CR1-/- mice. Unbiased Stereological counts for contralateral SNpc in WT and CX3CR1-/- mice.
(TIF)

## Abbreviations

PD: Parkinson disease
GWAS: genome wide association study
α-syn: alpha-synuclein
SNpc: substantia nigra pars compacta
HLA-DR: human leukocyte antigen
MHCII: major histocompatabillity complex II
TNF: tumor necrosis factor
IL-1β: interleukin 1 beta
IL-6: interleukin 6;
IFNg: interferon gamma
CX3CL1: fractalkine ligand
CX3CR1: fractalkine receptor
CNS: central nervous system
CX3CR1-/-: fractalkine receptor knockout
AAV: adeno-associated virus
GFP: green fluorescent protein
LPS: lipopolysaccharide
TH: tyrosine hydroxylase
DAB: diaminobenzadine
WT: wild type
IgG: immunoglobin G
MPTP: 1-methyl-4-phenyl-1,2,3,6-tetrahydropyridine
6-OHDA: 6-hydroxydopamine
CCL2: monocyte chemoattractant protein-1
