# Supplementary figures and images for: Fractalkine Signaling Regulates the Inflammatory Response in an α-Synuclein Model of Parkinson Disease
Source: PLoS One. 2015 Oct 15;10(10):e0140566. doi: 10.1371/journal.pone.0140566 (PMC4607155; doi:10.1371/journal.pone.0140566)

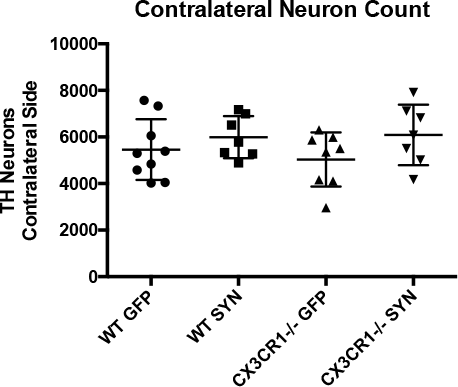

Supplement: S1 Fig — Unbiased Stereological counts for contralateral SNpc in WT and CX3CR1-/- mice. (TIF) [file pone.0140566.s001.tif]
